# Supplementary material for: Perception and knowledge of the effect of climate change on infectious diseases within the general public: A multinational cross-sectional survey-based study
Source: PLoS One. 2020 Nov 5;15(11):e0241579. doi: 10.1371/journal.pone.0241579 (PMC7644066; doi:10.1371/journal.pone.0241579)
Supplement: S1 File — (DOCX) [file pone.0241579.s001.docx]

**S1 File: Survey**

Q1: Please specify your age:  ………………….

Q2: What is your nationality? ………………….

Q3: What is your current country of residence? ………………….

Q4: Please specify your gender:

1. Male
2. Female
3. I prefer not to answer this question

Q5: Please specify your employment status:

1. Student
2. Full-time employed
3. Part-time employed
4. Unemployed/seeking for opportunities
5. Retired
6. Prefer not to say

Q6: My study or occupation is within the field of natural sciences (biology, medicine, public health):

1. Yes
2. No

Q7: What is the highest degree or level of school you have completed? *If currently studying, specify the degree you are enrolled in.*

1. High school (Spain: Instituto)
2. Vocational degree *(Nederlands: MBO) (Spain: Ciclo formativo)*
3. Bachelor’s degree *(Nederlands: HBO en WO bachelor) (Spain: grado universitario)*
4. Master’s degree
5. Doctoral degree

Q8: How many countries have you visited in the last 5 years (2015-now)?

1. 0 countries -  I have not been outside my country of residence
2. 1 - 3 countries
3. 4 - 6 countries
4. 7 - 10 countries
5. > 10 countries

Q9: How many continents have you visited in total?

1. 1 - I have never been outside my continent of residence
2. 2
3. 3
4. 4
5. 5
6. 6
7. 7

Q10: Have you ever lived in another country?

1. No
2. Yes, in another country but on the same continent
3. Yes, in another country which is on another continent

Q11: For each of the following statements, please select the most applicable one.

|  | Strongly  disagree | Disagree | Neutral | Agree | Strongly  agree | I don’t know |
| --- | --- | --- | --- | --- | --- | --- |
| 1, I consider myself as someone who knows more about infectious diseases than the general population |  |  |  |  |  |  |
| 2, I consider myself as someone who knows more about climate change than the general population |  |  |  |  |  |  |
| 3, I am well informed about about the effect of climate change on infectious diseases |  |  |  |  |  |  |
| 4, I believe that humans are responsible for global warming |  |  |  |  |  |  |
| 5, Climate change is going to be more severe in the future |  |  |  |  |  |  |
| 6, Global warming has already caused damage on human health |  |  |  |  |  |  |
| 7, I think that global warming has not yet affected humans, but will do so in the future |  |  |  |  |  |  |
| 8, Climate change has an effect on infectious diseases |  |  |  |  |  |  |
| 9, I am afraid of getting an infectious disease |  |  |  |  |  |  |
| 10, I am more afraid of getting an infectious disease if I am traveling to a tropical country |  |  |  |  |  |  |
| 11, Climate change & extreme weather conditions influence the spread of infectious diseases |  |  |  |  |  |  |
| 12, I always check if I need a vaccination before I go traveling to a tropical country |  |  |  |  |  |  |
| 13, It is important to research the effect of climate change on the spread of infectious diseases |  |  |  |  |  |  |
| 14, I have changed my behaviour in the recent years to minimise my impact on the environment |  |  |  |  |  |  |
| 15, I have changed my behaviour in the recent years to minimise the risk of getting an infectious disease |  |  |  |  |  |  |
| 16, Climate change will negatively impact our accessibility to food |  |  |  |  |  |  |
| 17, Global warming will mostly affect developing countries |  |  |  |  |  |  |
| 18, Research funding on climate change and infectious diseases should be increased |  |  |  |  |  |  |
| 19, I think that there will be more disease outbreaks in the future because of climate change |  |  |  |  |  |  |

Q12: Select all infectious diseases out of the following list:

- Malaria
- Tuberculosis
- Asthma
- Obesity
- Lyme disease
- Diabetes
- Allergies
- HIV

Q13: What risk factors are associated with getting an infectious disease?

Select all that apply:

- Insect bites
- Dog bites
- Smoking
- Drinking unclean water
- Eating undercooked food
- Not washing your hands often
- By sneezing
- Smelling farts
- Having sexual intercouse
- Sunbathing
- Breastfeeding
- Nove of the above

Q14: Insects that transmit diseases include // Disease transmission can occur via:

Please select all that apply:

- Mosquitoes *(Nederlands: muggen)*
- Sand flies *(Nederlands: zandvliegen)*
- Butterflies *(Nederlands: vlinders) (Spain: mariposas)*
- Head lice *(Nederlands: hoofdluis) (Spain: piojos)*
- Bed bugs *(Nederlands: bedmijt) (Spain: chinches de cama)*
- Black flies *(Nederlands: zwarte vliegen) (Spain: moscas negras)*
- Ticks *(Nederlands: teek) (Spain: garrapatas)*
- Wasps *(Nederlands: wespen) (Spain: avispas)*
- Fleas *(Nederlands: vlooien) (Spain: pulgas)*
- Body lice *(Nederlands: lichaamsluizen) (Spain: ladillas)*
- Dogs *(Spain: perro)*
- Cats *(Spain: gato)*
- Horses *(Spain: caballo)*
- Nove of the above

Q15: For each of the following statements, please select the most applicable one.

|  | True | False | I don’t know |
| --- | --- | --- | --- |
| 1, Infectious diseases affect both humans and animals |  |  |  |
| 2, Malaria can be acquired by being close to farm animals |  |  |  |
| 3, Dengue and Zika are transmitted by mosquitoes |  |  |  |
| 4, Infectious diseases can be spread via water |  |  |  |
| 5, Antibiotics can be used to treat several viral diseases, such as influenza and measles |  |  |  |
| 6, Hand washing can stop the spread of infectious diseases |  |  |  |
| 7, Vaccines can be used to prevent bacterial diseases |  |  |  |

Q16: For each of the following statements, please select the most applicable one.

|  | True | False | I don’t know |
| --- | --- | --- | --- |
| 1, Climate change has an effect on global temperatures |  |  |  |
| 2, Climate change has an impact on rainfall |  |  |  |
| 3, The greenhouse effect is the process by which gases in the atmosphere that trap heat |  |  |  |
| 4, The greenhouse effect is caused by some gases that heat the atmosphere |  |  |  |
| 5, Increased carbon dioxide levels are a cause of climate change |  |  |  |
| 6, The burning of fossil fuels is a major contributor to global warming |  |  |  |
| 7, Only developed countries are responsible of the greenhouse gas emissions |  |  |  |
| 8, Over the past 100 years, the rate of melting of glaciers has increased |  |  |  |
| 9, Climate change will reduce the frequency and severity of natural disasters |  |  |  |
| 10, Climate change causes the displacement of people |  |  |  |
| 11, Climate change is worsened by carbon dioxide emissions |  |  |  |
| 12, Intensive farming contributes to climate change. |  |  |  |
| 13, Vegetable and fruit consumption **does not** contribute to climate change |  |  |  |
| 14, Climate change mainly affects developed countries |  |  |  |
| 15, The climate is always changing. It’s natural. |  |  |  |

Q17: For each of the following statements, please select the most applicable one.

|  | Yes | No | I don’t know |
| --- | --- | --- | --- |
| 1, Mosquitoes survive better at warmer temperatures |  |  |  |
| 2, The chance of getting lyme disease in Europe will not be increased as an effect of global warming. |  |  |  |
| 3, There will be more tick activity if the temperature is increasing in colder countries (such as Canada and Sweden) |  |  |  |
| 4, Mosquito-borne diseases (such as malaria) will become a bigger issue in countries with a cooler climate (such as Europe and the US) if the temperature increases |  |  |  |
| 5, Higher rainfall will impact infectious diseases |  |  |  |
| 6, There is **no** direct link between climate change and infectious disease outbreaks |  |  |  |
| 7, Floods can increase the number of outbreaks of infectious diseases |  |  |  |
| 8, Climate change has an impact on infectious diseases that are not transmitted by mosquitoes or ticks |  |  |  |
| 9, Climate change has an impact on viral diseases |  |  |  |
| 10, Climate change does not have an impact on bacteriological diseases |  |  |  |
| 11, Temperature has an influence on the survival of virus or bacteria in the environment |  |  |  |
| 12, The infectious diseases that affect animals are not influenced by the weather |  |  |  |
| 13, Droughts can favour the transmission of infectious diseases |  |  |  |
| 14, Climate change can reduce the transmission of some infectious diseases |  |  |  |
| 15, Climate change will not affect the transmissibility of infectious diseases in countries which already have high temperatures. |  |  |  |

Q18: Before this survey, did you consider the impact of climate change on infectious diseases in your daily life and in your environment?

1. Yes
2. No

Q19: Has this survey changed your opinion on this topic?

1. Yes
2. No

Q20: Please specify one information source you have obtained the most knowledge from regarding the impact of climate change on infectious diseases:

1. Internet and social media
2. Websites of governments and official institutions
3. Television programs and documentaries
4. Books and newspapers
5. Radio and podcasts
6. Family and friends
7. Educational programs and conferences

Q21: Would you like to learn more about this topic?

1. Yes
2. No
3. I don’t care / Indifferent
