# Supplementary material for: Perception and knowledge of the effect of climate change on infectious diseases within the general public: A multinational cross-sectional survey-based study
Source: PLoS One. 2020 Nov 5;15(11):e0241579. doi: 10.1371/journal.pone.0241579 (PMC7644066; doi:10.1371/journal.pone.0241579)
Supplement: S2 File — (DOCX) [file pone.0241579.s002.docx]

**S2 File: Answer key**

|  | True | False | I don’t know |
| --- | --- | --- | --- |
| **Identification of infectious diseases (Q12)** | | | |
| Malaria | + 1 | - 0.5 |  |
| Tuberculosis | + 1 | - 0.5 |  |
| Asthma | - 0.5 | + 1 |  |
| Obesity | - 0.5 | + 1 |  |
| Lyme disease | + 1 | - 0.5 |  |
| Diabetes | - 0.5 | + 1 |  |
| Allergies | - 0.5 | + 1 |  |
| HIV | + 1 | - 0.5 |  |
| **Identification of risk factors of getting an infectious disease (Q13)** | | | |
| Insect bites | + 1 | - 0.5 |  |
| Dog bites | + 1 | - 0.5 |  |
| Smoking | - 0.5 | + 1 |  |
| Drinking unclean water | + 1 | - 0.5 |  |
| Eating undercooked food | + 1 | - 0.5 |  |
| Not washing your hands often | + 1 | - 0.5 |  |
| By sneezing | + 1 | - 0.5 |  |
| Smelling farts | - 0.5 | + 1 |  |
| Having sexual intercourse | + 1 | - 0.5 |  |
| Sunbathing | - 0.5 | + 1 |  |
| Breastfeeding | + 1 | - 0.5 |  |
| None of these above | 0 | 0 |  |
| **Identification of animals that can transmit infectious diseases (Q14)** | | | |
| Mosquitoes | + 1 | - 0.5 |  |
| Sand flies | + 1 | - 0.5 |  |
| Butterflies | - 0.5 | + 1 |  |
| Head lice | - 0.5 | + 1 |  |
| Bed bugs | - 0.5 | + 1 |  |
| Black lies | + 1 | - 0.5 |  |
| Ticks | + 1 | - 0.5 |  |
| Wasps | - 0.5 | + 1 |  |
| Fleas | + 1 | - 0.5 |  |
| Body lice | + 1 | - 0.5 |  |
| Dogs | + 1 | - 0.5 |  |
| Cats | + 1 | - 0.5 |  |
| Horses | + 1 | - 0.5 |  |
| None of these above | 0 | 0 |  |
| **Knowledge assessment on infectious diseases** | | | |
| Q15.1: Infectious diseases affect both humans and animals | + 1 | - 0.5 | 0 |
| Q15.2: Dengue and Zika are transmitted by mosquitoes | + 1 | - 0.5 | 0 |
| Q15.3: Infectious diseases can be spread via water | + 1 | - 0.5 | 0 |
| Q15.4: Antibiotics can be used to treat several viral diseases, such as influenza and measles | - 0.5 | + 1 | 0 |
| Q15.5: Hand washing can stop the spread of infectious diseases | + 1 | - 0.5 | 0 |
| Q15.6: Vaccines can be used to prevent bacterial diseases | + 1 | - 0.5 | 0 |
|  |  |  |  |
|  | True | False | I don’t know |
| **Knowledge assessment on climate change** | | | |
| Q16.1: Climate change has an effect on global temperatures | + 1 | - 0.5 | 0 |
| Q16.2: Climate change has an impact on rainfall | + 1 | - 0.5 | 0 |
| Q16.3: The greenhouse effect is the process by which gases in the atmosphere that trap heat | + 1 | - 0.5 | 0 |
| Q16.4: The greenhouse effect is caused by some gases that heat the atmosphere | - 0.5 | + 1 | 0 |
| Q16.5: Increased carbon dioxide levels are a cause of climate change | + 1 | - 0.5 | 0 |
| Q16.6: The burning of fossil fuels is a major contributor to global warming | + 1 | - 0.5 | 0 |
| Q16.7: Only developed countries are responsible of the greenhouse gas emissions | - 0.5 | + 1 | 0 |
| Q16.8: Over the past 100 years, the rate of melting of glaciers has increased | + 1 | - 0.5 | 0 |
| Q16.9: Climate change will reduce the frequency and severity of natural disasters | - 0.5 | + 1 | 0 |
| Q16.10: Climate change causes the displacement of people | + 1 | - 0.5 | 0 |
| Q16.11: Climate change is worsened by carbon dioxide emissions | + 1 | - 0.5 | 0 |
| Q16.12: Intensive farming contributes to climate change | + 1 | - 0.5 | 0 |
| Q16.13: Vegetable and fruit consumption does not contribute to climate change | - 0.5 | + 1 | 0 |
| Q16.14: Climate change mainly affects developed countries | - 0.5 | + 1 | 0 |
| Q16.15: The climate is always changing. It’s natural | + 1 | - 0.5 | 0 |
| **Knowledge assessment on the effect of climate change on infectious diseases** | | | |
| Q17.1: Mosquitoes survive better at warmer temperatures | + 1 | - 0.5 | 0 |
| Q17.2: There will be more tick activity if the temperature is increasing in colder countries (such as Canada and Sweden) | + 1 | - 0.5 | 0 |
| Q17.3: Mosquito-borne diseases (such as malaria) will become a bigger issue in countries with a cooler climate (such as Europe and the US) if the temperature increases | + 1 | - 0.5 | 0 |
| Q17.4: Higher rainfall will impact infectious diseases | + 1 | - 0.5 | 0 |
| Q17.5: There is no direct link between climate change and infectious disease outbreaks | - 0.5 | + 1 | 0 |
| Q17.6: Floods can increase the number of outbreaks of infectious diseases | + 1 | - 0.5 | 0 |
| Q17.7: Climate change has an impact on infectious diseases that are not transmitted by mosquitoes or ticks | + 1 | - 0.5 | 0 |
| Q17.8: Climate change has an impact on viral diseases | + 1 | - 0.5 | 0 |
| Q17.9: Climate change does not have an impact on bacteriological diseases | - 0.5 | + 1 | 0 |
| Q17.10: Temperature has an influence on the survival of virus or bacteria in the environment | + 1 | - 0.5 | 0 |
| Q17.11: The infectious diseases that affect animals are not influenced by the weather | - 0.5 | + 1 | 0 |
| Q17.12: Droughts can favour the transmission of infectious diseases | + 1 | - 0.5 | 0 |
| Q17.13: Climate change can reduce the transmission of some infectious diseases | + 1 | - 0.5 | 0 |
| Q17.14: Climate change will not affect the transmissibility of infectious diseases in countries which already have high temperatures. | - 0.5 | + 1 | 0 |
