# Supplementary material for: Perception and knowledge of the effect of climate change on infectious diseases within the general public: A multinational cross-sectional survey-based study
Source: PLoS One. 2020 Nov 5;15(11):e0241579. doi: 10.1371/journal.pone.0241579 (PMC7644066; doi:10.1371/journal.pone.0241579)

**S3 File: Scoring key**

***Scoring scheme for each assessment***

Each response was recorded as +1 for a correct answer, -0.5 for an incorrect answer and 0 for *I don’t know*. The scoring key can be found in file S2. Three topics were assessed in the current study: knowledge on infectious diseases, climate change and the effect of climate change on infectious diseases.

The maximum score for the three different knowledge assessments have been normalized to 20 in order to have a more consistent score and making it easier to compare.

Score of climate change was adjusted from 15 to 20 (x 1.33)

Score of effect of climate change on infectious diseases was adjusted from 14 to 20 (x 1.43)

**Table S1: Scoring key for knowledge assessments.** The three topics assessed in the current study were knowledge on infectious diseases, climate change and the effect of climate change on infectious diseases. Individuals scores were grouped with respect to the maximum possible score and marked according to the American grading system as displayed below. The maximum score for each separate knowledge assessment has been normalized to 20.


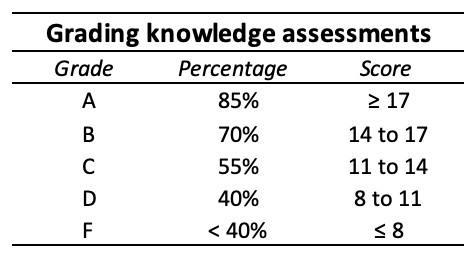


***Scoring scheme for knowledge assessment on infectious diseases***

The score for Infectious Diseases has been composed of four different aspects, which all weighted for 25% of the total score. The scores for all knowledge assessments were normalized to a maximums core of 20.

**Table S2: Separate scoring key for the knowledge assessment on infectious diseases.** The score for infectious diseases was composed of four different questions of the survey, which all weighted for 25% of the final score. The performed calculation is visualized below.


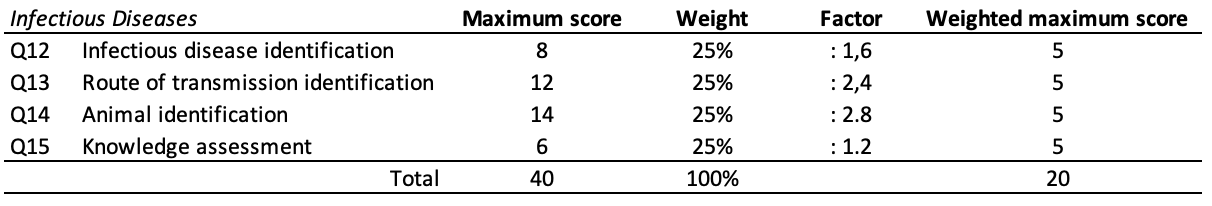

Supplement: S3 File — (DOCX) [file pone.0241579.s003.docx]
