# Supplementary figures and images for: Perception and knowledge of the effect of climate change on infectious diseases within the general public: A multinational cross-sectional survey-based study
Source: PLoS One. 2020 Nov 5;15(11):e0241579. doi: 10.1371/journal.pone.0241579 (PMC7644066; doi:10.1371/journal.pone.0241579)

**S4 File: Demographic overview of the top 5 represented countries**


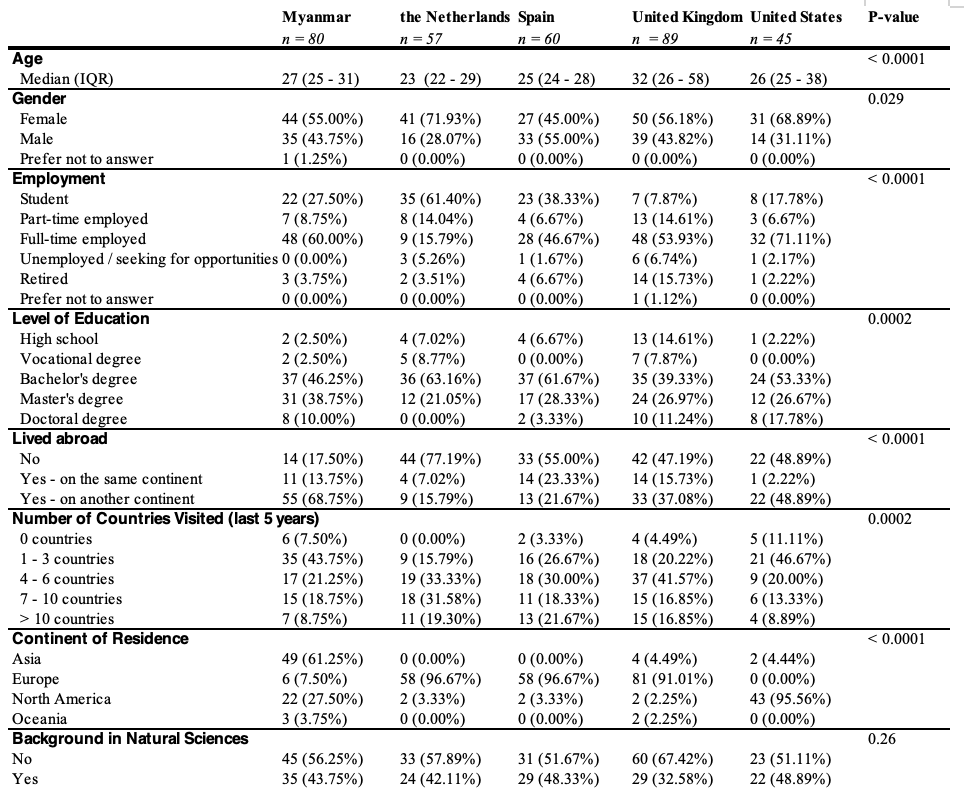

Supplement: S4 File — (DOCX) [file pone.0241579.s004.docx]
