# Supplementary material for: Perception and knowledge of the effect of climate change on infectious diseases within the general public: A multinational cross-sectional survey-based study
Source: PLoS One. 2020 Nov 5;15(11):e0241579. doi: 10.1371/journal.pone.0241579 (PMC7644066; doi:10.1371/journal.pone.0241579)
Supplement: Attachment — Submitted filename: Response to Reviewers.docx [file pone.0241579.s005.docx]

5. Review Comments to the Author

Please use the space provided to explain your answers to the questions above. You may also include additional comments for the author, including concerns about dual publication, research ethics, or publication ethics. (Please upload your review as an attachment if it exceeds 20,000 characters)

Reviewer #1: Van Wijk and colleagues have performed an interesting survey-based study to investigate the knowledge of the participants on 1) climate change, 2) infectious disease and 3) the effect of climate change on infectious disease. Participants were split primarily in to the general public with and without a background in natural science and by nationality.

Minor points:

It would be interesting to further dissect those participants that did not have a background in natural science based on their occupation. i.e did those participants with higher skilled jobs have a better understanding? This may further reflect the education of the participants. We agree with the reviewer that it would have been interesting to analyse this information but unfortunately, we did not collect this information in the survey we spread. Therefore, this information cannot be provided and included in the manuscript. We have included a sentence indicating this aspect in the section “Limitations and future implications” of the manuscript.

Further, it would be interesting to investigate the level of education (which it was highlighted that these data were collected) on the knowledge of 1) climate change, 2) infectious disease and 3) the effect of climate change on infectious disease. This may identify were best to target education for specific groups to better inform the general public on infectious disease and/or climate change. This information has been analysed and integrated in the section “Knowledge assessment”. As indicated in the text, the level of education was positively associated with level of knowledge only in the case of “climate change”.

Reviewer #2: The manuscript is interesting, the topic is actual and relevant – infectious diseases, climate changes and the interactions between both, within a general public.
Being a multinational cross sectional survey-based study it allows comparisons among results from different places and persons.
The questionnaire adopted is adequate, well written, concerning:
- personal data (Q1-Q10)
- knowledge in infectious diseases (Q12- Q16.5)
- knowledge on climate change (Q16.1 – Q16.15)
- knowledge on the effect of climate change on infectious disease (Q17.1-Q17.14)

The population studied is diverse, the cohort size adequate.
The data transformation and grading is adequate and allows an easy data interpretation.
The results are well presented and the discussion is easy to follow
Weakness of the work are identified, and I would say that discussion should be slight shortened (6 pages). Discussion has been shortened to 6 pages, as suggested by the reviewer.
